# Plasma membrane partitioning: from macro-domains to new views on plasmodesmata

**DOI:** 10.3389/fpls.2014.00128

**Published:** 2014-04-03

**Authors:** Yohann Boutté, Patrick Moreau

**Affiliations:** CNRS, Laboratoire de Biogenèse Membranaire, UMR 5200, Université Bordeaux Segalen, INRA Bordeaux AquitaineVillenave d’Ornon, France

**Keywords:** domains partitioning, lateral diffusion, GTPases, PIN proteins, plasma membrane recycling, endocytosis, lipids, casparian strip

## Abstract

Compartmentalization of cellular functions relies on partitioning of domains of diverse sizes within the plasma membrane (PM). Macro-domains measure several micrometers and contain specific proteins concentrated to specific sides (apical, basal, and lateral) of the PM conferring a polarity to the cell. Cell polarity is one of the driving forces in tissue and growth patterning. To maintain macro-domains within the PM, eukaryotic cells exert diverse mechanisms to counteract the free lateral diffusion of proteins. Protein activation/inactivation, endocytosis, PM recycling of transmembrane proteins and the role of diffusion barriers in macro-domains partitioning at PM will be discussed. Moreover, as plasmodesmata (PDs) are domains inserted within the PM which also mediate tissue and growth patterning, it is essential to understand how segregation of specific set of proteins is maintained at PDs while PDs domains are smaller in size compared to macro-domains. Here, we will present mechanisms allowing restriction of proteins at PM macro-domains, but for which molecular components have been found in PDs proteome. We will explore the hypothesis that partitioning of macro-domains and PDs may be ruled by similar mechanisms.

## INTRODUCTION

A fascinating feature of living organisms is their ability to compartmentalize functions which from the sub-cellular level organize the entire organism. Similarly, to the specialization of intracellular functions in diverse organelles inside the cell, lateral compartmentalization of the plasma membrane (PM) organizes cell functions by spatially restricting interactions between specific sets of proteins and between proteins and specific membrane lipids to defined areas of the PM. Large scale domains segregation (e.g., several micrometers macro-domains) within the PM (basal, apical, outer lateral, and inner lateral membranes) is the basis for cell polarity which in turn will determine growth patterns in the whole organism. Plants have the ability to reorient the pattern of their body plan throughout the entire life span and are thus highly flexible in modulating cell polarity and segregating domains at the PM. Although our understanding of the mechanisms involved in lateral heterogeneity of the PM remains fragmented, recent progress has been made in various model systems. Here, we will review and discuss sub-cellular and molecular mechanisms which take an active part in lateral segregation of macro-domains within the PM. In a first part of this review we will describe a protein activation/inactivation mechanism through which polar domains, established at the tip of plants pollen tube cells and root hair cells, conduct polar tip growth of the cell. In a second part, we will focus on regulations acting on recycling and defined endocytosis at PM polar domains and how that is restricting lateral mobility of proteins at specific domains at PM. In a third part we will succinctly present membrane lipids as major regulators of PM macro-domains partitioning. In a fourth part, we will present recent progress made in the comprehension of the role of diffusion barriers, such as the endodermal casparian strip, and the extracellular matrix in segregating macro-domains at the PM. At last we will discuss the specific case of smaller scale PM domains than macro-domains but which function is crucial in tissue identity and patterning, e.g., the plasmodesmata (PDs). We expect that mechanisms known from other models than PDs, but for which molecular elements have been found in the plasmodesmal proteome, could help understanding proteins segregation at PDs.

## REGULATION OF PROTEIN ACTIVATION PLAYS A ROLE IN LATERAL SEGREGATION OF PROTEINS AT THE PM

Guanosine triphosphate (GTP)-binding proteins are involved in determining specificity of membrane fusion and fission events. Therefore, lateral segregation of these proteins at the PM must be under fine tuned control to ensure that specific trafficking events act at defined spots. Lateral segregation of GTP-binding proteins is partly regulated through activation and inactivation of these GTP switches. Guanine nucleotide exchange factors (GEFs) catalyze the conversion of the GTP-binding protein from the inactive guanosine diphosphate (GDP)-bound form to the active GTP-bound form. Conversely, GTPase-activating proteins (GAPs) stimulate hydrolysis of GTP to GDP. Moreover, binding of guanosine nucleotide dissociation factors (GDIs) to the inactive GDP-bound form prevents GDP to GTP exchange. To be activated, proteins bound to GDP must be released from their GDI inhibitor through action of GDI displacement factors. Conversion in the active state favors interactions with downstream effectors involved in diverse cellular functions such as polar growth for example (Molendijk et al., 2001, 2004; Vernoud et al., 2003). Some plant RHO-type GTP-binding proteins have been shown to preferentially locate in a polar fashion in polarized tip growing cells such as pollen tubes and root hairs (Lin et al., 1996; Jones et al., 2002). Both GDI and GAPs proteins have been shown to be involved in the polar localization of RHO proteins at the tip of apically growing cells. In *Arabidopsis* root hairs, the RHO protein ROP2 is polarized to the tip of the cell through a mechanism in which the GDI protein SUPERCENTIPEDE1 (SCN1)/AtRHO-GDI1 plays a major function (Carol et al., 2005). This illustrates the role of the inactivation of RHO in the regulation of its activity and its restricted polarized localization. Consistently, in tobacco pollen tubes, the GDI protein NtRHO-GDI2 and the GAP protein NtRHO-GAP1 spatially restricts the RHO-type GTP-binding protein NtRAC5 activity to the tip of pollen tubes (Klahre and Kost, 2006; Klahre et al., 2006). In this model, NtRHO-GAP1 localizes at the lateral PM close to the tip of the pollen tube but is excluded from the very tip where active membrane-bound NtRAC5 is found (Klahre and Kost, 2006). Lateral diffusion of active NtRAC5 to lateral PM would then be counteracted by inactivation of NtRAC5 which would then dissociate from the PM. However, this mechanism is not a general mechanism since in *Arabidopsis* pollen tubes the RHO-GAP protein REN1 localizes to the tip of pollen tube where the RHO-type GTP-binding protein ROP1 is present (Hwang et al., 2008). In this model, when ROP1 reaches a critical threshold level at the PM, REN1 initiates a negative feedback loop to inactivate ROP1 which would be removed from the membrane. The cyclic nature of this phenomenon results in an oscillatory tip growth (Hwang et al., 2008). If lateral segregation of membrane associated GTP-binding proteins is regulated through GTP/GDP switch, this mechanism however, does not explain how intrinsic transmembrane proteins can be confined to specific domains of the PM.

## ENDOCYTOSIS AND RECYCLING ARE INVOLVED IN SPATIAL SEGREGATION OF PROTEINS AT THE PM BY RESTRICTING LATERAL DIFFUSION

Lateral segregation of auxin carriers in the PM of *Arabidopsis* roots is the most extensively studied model to understand how cell polarity of transmembrane proteins is established and maintained in distinct PM macro-domains such as apical, basal, inner lateral, and outer lateral membranes. Polar localization of the auxin efflux carriers PINs at the basal membrane greatly relies on endocytosis and PM recycling. PIN recycling has been shown to involve ADP-ribosylation factor (ARF) activation through the ARF-GEF GNOM which localizes to recycling endosomes which have yet to be structurally defined (Steinmann et al., 1999; Geldner et al., 2003; Richter et al., 2007). Interestingly, another ARF-GEF, BEN1, has been shown to act in PIN recycling at early endocytic compartments distinct from GNOM-labeled endosomes pointing out a differential regulation on PIN recycling (Tanaka et al., 2009). Additionally, ARF inactivation through the ARF-GAP SCARFACE (SFC)/VAN3 also plays a crucial function in the PM recycling of PINs (Sieburth et al., 2006). Targets of ARF-GEF and ARF-GAP are not well described although it is known that the ARF-GEF BIG3 interacts *in vitro* with ARF1-A1C protein required for BFA-sensitive PM recycling of PIN proteins (Nielsen et al., 2006; Tanaka et al., 2014). It is thought that ARF-GEF membrane recruitment to specific compartments is partly regulated through the action of RAB proteins. Consistently, it has been shown that the RAB-A1b and RAB-A1c proteins are involved in lateral segregation of PINs proteins through PM recycling (Qi et al., 2011; Feraru et al., 2012). Moreover, effector elements of RAB molecular switches are also involved. For example, subunits of the exocyst vesicle tethering complex, thought to be RAB effectors and to act in polarization of exocytosis in yeast and animals, are involved in the recycling of PIN proteins (Drdova et al., 2013). Together with PM recycling, computer simulations have suggested that PIN polar domain is maintained through the occurrence of a spatially defined clathrin-mediated endocytic area at the circumventing edges of the polar domain (Kleine-Vehn et al., 2011). During endocytosis of PIN proteins, clathrin-coated vesicles (CCVs) form at the PM and loading of cargoes in these vesicles is selectively occurring via clathrin adaptor complexes while fission of CCVs from the PM is promoted by dynamin-related proteins (Dhonukshe et al., 2007; Fujimoto et al., 2010; Kitakura et al., 2011; Mravec et al., 2011; Fan et al., 2013). Additionally, RHO-type GTP-binding proteins ROP2 and ROP6 are master regulators of clathrin-dependent endocytosis pointing out again the central importance of GTP-binding proteins in lateral segregation of proteins (Chen et al., 2012; Lin et al., 2012; Nagawa et al., 2012).

## MEMBRANE LIPIDS ARE MAJOR REGULATORS OF PM MACRO-DOMAINS PARTITIONING

Several classes of lipids have been shown to be implicated in partitioning of macro-domains at PM. Bulk sterols are membrane lipids and key elements in endocytosis-mediated establishment of the auxin efflux carrier PIN2 polar domains at the PM during post-cytokinesis (Men et al., 2008). Recently, it has been shown that not only bulk sterols are involved in polar auxin transport but sterol biosynthetic intermediates also act in this process pointing out the likely high complexity of sterols in regulation of polarity of auxin carriers (Mialoundama et al., 2013). Additionally, sterol-mediated endocytosis is involved, together with clathrin-mediated endocytosis, in restricting lateral diffusion of a major player of vesicle fusion, the syntaxin KNOLLE, from the division plane to lateral membranes during cytokinesis (Boutté et al., 2010). Accordingly, clathrin light chain and one of its interacting partners, T-PLATE, accumulate at the cortical division zone of lateral PM juxtaposing the division plane (Van Damme et al., 2011). These studies suggest that spatially defined sterol- and clathrin-mediated endocytosis regulate lateral segregation of proteins to the division plane during cytokinesis. Very-long-chain-fatty-acids (VLCFAs) present in phospholipids and sphingolipids pools were also shown to be important in endocytosis and restriction of the KNOLLE macro-domain at the cell plate during cytokinesis (Bach et al., 2011). Indeed, it was also shown previously that an isoform of the phospholipase D, which cleaves the phosphodiester bound of a phospholipid and consequently produces the phosphatidic acid (PA), and an isoform of the phospholipase A, which cleaves a fatty acyl chain of a phospholipid, are critical for endocytosis and recycling at PIN2 PM macro-domain (Li and Xue, 2007; Lee et al., 2010). Sphingolipids are also known to be involved in polarized PM distribution of PIN although the precise role played by sphingolipids in cellular mechanisms, e.g., secretory and endocytic pathways, still has to be determined (Roudier et al., 2010; Markham et al., 2011). Lastly, phosphoinositides, although representing a minor class of phospholipids, also appear as a crucial class of lipids involved in segregation of PIN polar domains at the PM. This is exemplified recently by the role of phosphatidylinositol 4,5-biphosphate [PtdIns(4,5)P_2_] in clathrin-mediated PIN1 and PIN2 polar domains segregation at the PM (Ischebeck et al., 2013). Additionally to endomembrane trafficking events and their regulation, the extracellular environment of the cell has also to be considered in segregating domains at PM. Indeed, the cell wall plays an integral role in the framework modulating cell polarity and lateral diffusion of proteins (Feraru et al., 2011; Martinière et al., 2012).

## MECHANICAL CONSTRAINTS APPLIED BY THE EXTRACELLULAR MATRIX INFLUENCE LATERAL SEGREGATION OF PROTEINS IN PM

Eukaryotic multicellular organisms such as animals and plants are using the extracellular matrix properties to segregate domains within the PM. In polarized animal cells, junctional complexes (including tight junctions and adherens junctions) are known to be involved in restricting the movement of membrane proteins and lipids (van Meer and Simons, 1986; Laval et al., 2008; Renner et al., 2012). Junctions are constituted of a dense scaffolded arrangement of proteins included in the extracellular matrix made of polysaccharides. In plants, the cell wall is also constituted of polysaccharides such as cellulose (polymer of D-glucose through β-1,4-linkage), hemicelluloses (xyloglucan and xylan, a polymere of xylose), pectins (homogalacturonans and substituted galacturonans), and lignin (phenolic polymer). A good example of PM domains segregation mediated by cell wall specialization is the casparian strip, a belt which transversally surrounds endodermal root cells in an anticlinal orientation and which is constituted of lignin in the early stages, although suberin could also be constitutive at later stages (Alassimone et al., 2010; Naseer et al., 2012). The casparian strip is a diffusion barrier which functional features resemble tight and adherens junctions of animal polarized epithelia although protein and extracellular matrix composition is different (Roppolo et al., 2011; Naseer et al., 2012). The casparian strip is splitting endodermal cells in distinct lateral PM domains, an outer domain facing the periphery of the root and an inner domain facing the central part of the root in which distinct proteins are found (Alassimone et al., 2010). The PM domain underlying the casparian strip is located at the crossroad of the outer and inner lateral domains and contains casparian strip domain proteins (CASPs) which constitute a dense scaffold and are involved in focalizing lignin deposition during casparian strip formation (Roppolo et al., 2011; Lee et al., 2013). CASPs proteins and the specialized cell wall region of the casparian strip form a diffusion barrier for proteins and lipids (Alassimone et al., 2010). Segregation of lateral PM domains allows endodermal cells to select nutrients and minerals as exemplified with the boron influx transporter NIP5;1 which localization is constrained to the outer lateral domain and the boron efflux transporter BOR1 which restriction to the inner lateral domain consistently supports its role in loading xylem cells of root central cylinder (Alassimone et al., 2010; Takano et al., 2010). Additionally, localization of other carriers such as auxin carriers is also differentially regulated in endodermal cells in which the casparian strip is present. The auxin efflux carrier PIN2 is found to be localized at the basal membrane in protoendodermal cells, at the apical membrane in elongating endodermal cells and finally at the inner lateral membrane in differentiated endodermal cells in which the casparian strip is in place (Alassimone et al., 2010). This cell differentiation stage-dependent relocation of PIN2 exemplifies that regulation of domain segregation within the PM can be different when a diffusion barrier such as the casparian strip is present.

The cell wall is also known to be involved in membrane domains segregation in other cell types than differentiated endodermal cells. In BY2 cells and *Arabidopsis* meristematic root cells, it has been shown that PIN polarity is lost when the cell wall is progressively chemically removed (Boutté et al., 2006; Feraru et al., 2011). Moreover, mutants deficient in cellulose composition display PIN polarity defects at the PM of epidermal cells (Feraru et al., 2011). Interestingly, a gentle plasmolysis which preserves the cell wall results in retraction of the PM but retains PM connection with the cell wall at defined spots labeled with PM proteins localized in polar domains, such as the auxin efflux carrier PIN1, but not with proteins which localization is apolar (Feraru et al., 2011). This observation suggests that specific membrane attachment to the extracellular matrix could be important in lateral segregation of domains.

## SEGREGATION OF PLASMODESMATA WITHIN THE PM COULD SHARE SIMILAR MECHANISMS AS FOR MACRO-DOMAINS

Plasmodesmata are specific domains of the PM organized as nanopores and which function in cell-to-cell communication is essential to virus movement, cell identity induced by movement of transcription factors from cell layer to cell layer, and patterning during lateral roots development and embryogenesis (Wolf et al., 1989; Lucas et al., 1995; Kim et al., 2005; Kurata et al., 2005; Vaten et al., 2011; Wu and Gallagher, 2012; Benitez-Alfonso et al., 2013; Tilsner et al., 2013). Hence, given their role in diverse processes, it is essential to understand how plants restrict proteins at PDs sites of the PM. Whether regulation of protein activation through GDP/GTP cycling has a function in restricting lateral diffusion of proteins at PDs remain unaddressed. However, several GTP-binding proteins are found in the *Arabidopsis* plasmodesmal proteome: proteins from the ARF family (all from the subclass A, and several ARF-like proteins), proteins from the RAB family (from subclasses A, E, and G) and proteins from the GDI and GEF families (Fernandez-Calvino et al., 2011). GTP-binding proteins of the dynamin-related family which could be involved in membrane constriction of the desmotubulus were also found and their localization could also be regulated through GTP/GDP switch. Moreover, PDs are involved in movement of viruses and a recent study suggests a role for a RAB-GAP in Bamboo mosaic virus intercellular movement (Huang et al., 2013). Alternatively and/or additionally, GTP-binding proteins could be involved in PDs-localized endocytosis and recycling which would restrict lateral diffusion of proteins at PDs. Indeed, elements of the clathrin machinery have been identified in the plasmodesmal proteome including clathrin heavy chain, subunits of clathrin adaptor complexes, and dynamin-related proteins (Fernandez-Calvino et al., 2011). Apart from proteins, the function of membrane lipids in lateral segregation of proteins at PDs, and in PDs functioning in general, is still a determinant question to address. Similarly, the function of the cell wall in lateral segregation of proteins at PDs is still to be addressed. Interestingly, PDs are structures which retain PM close contact to the cell wall upon plasmolysis similarly to what has been described for PIN1 (Oparka, 1994; Feraru et al., 2011). Interestingly, auxin efflux carriers PIN1 and PIN7 have been found in the plasmodesmal proteome (Fernandez-Calvino et al., 2011). Hence, it is possible that specific spots where PINs labeled membranes attached to the cell wall correspond to PDs. However, it is not known whether cell wall components known to be involved in lateral segregation of polar domains, such as cellulose, play a role in PDs functioning and/or lateral segregation of domains at PDs, although cellulose synthases have been found in the plasmodesmal proteome (Fernandez-Calvino et al., 2011).

## CONCLUSION

Eukaryotic cells partition the PM into distinct domains to compartmentalize cellular processes and specify functions critical in growth patterning. Polar tip growth and auxin-mediated developmental processes require segregation of polar domains at the PM. Protein activation/inactivation, PM recycling and endocytosis, diffusion barriers, and the extracellular matrix are known to be involved in domain partitioning during polar tip growth or in auxin carriers polar positioning at the PM. PDs which function is crucial in tissue patterning also requires partitioning of proteins. This specifies domains and segregate processes although mechanisms through which this happens have yet to be discovered. Future studies might reveal whether and how plasmodesmal-localized activable GTP-binding proteins, and their regulators, are involved in lateral segregation of proteins involved in plasmodesmal-related processes. Importantly, whether endocytosis and recycling are also involved in lateral segregation at PDs should be investigated. Finally, as PDs can be viewed as cell junctions, future studies would need to combine genetics, biochemistry, and cell biology to decipher the role of cell wall components in the function and lateral segregation of proteins at PDs.

## Conflict of Interest Statement

The authors declare that the research was conducted in the absence of any commercial or financial relationships that could be construed as a potential conflict of interest.
